# Supplementary material for: Comprehensive evolutionary analysis and nomenclature of plant G3BPs
Source: Life Sci Alliance. 2022 May 26;5(9):e202101328. doi: 10.26508/lsa.202101328 (PMC9136153; doi:10.26508/lsa.202101328)
Supplement: Supplementary file 4 [file LSA-2021-01328_TableS1.docx]

Supplementary Table 1.

| category | species | proteins |
| --- | --- | --- |
| Dicots | *Arabidopsis thaliana* | GCF_000001735.4 |
|  | *Solanum lycopersicum* | GCF_000188115.4 |
|  | *Eucalyptus grandis* | GCF_016545825.1 |
|  | *Medicago truncatula* | GCF_003473485.1 |
|  | *Populus trichocarpa* | GCF_000002775.4 |
|  | *Citrus sinensis* | GCF_000317415.1 |
|  | *Theobroma cacao* | GCF_000208745.1 |
|  | *Brassica oleracea* | GCF_000695525.1 |
|  | *Aquilegia coerulea* | GCA_002738505.1 |
| Monocots | *Oryza sativa* | GCF_001433935.1 |
|  | *Zea mays* | GCF_902167145.1 |
|  | *Setaria italica* | GCF_000263155.2 |
|  | *Ananas comosus* | GCF_001540865.1 |
|  | *Musa acuminata* | GCF_000313855.2 |
|  | *Oropetium thomaeum* | Othomaeum_386_v1.0 |
|  | *Zostera marina* | GCA_001185155.1 |
| Basal Angiosperm | *Amborella trichopoda* | GCF_000471905.2 |
| Mosses | *Physcomitrella patens* | GCF_000002425.4 |
|  | *Sphagnum fallax* | GCA_021442195.1 |
| Marchantiophyta | *Marchantia polymorpha* | GCA_003032435.1 |
| Metazoans | *Homo sapiens* | GCF_000001405.39 |
|  | *Xenopus tropicalis* | GCF_000004195.4 |
|  | *Danio rerio* | GCF_000002035.6 |
|  | *Ciona savignyi* | Ciona_savignyi.CSAV2.0 |
|  | *Strongylocentrotus purpuratus* | GCF_000002235.5 |
|  | *Drosophila melanogaster* | GCF_000001215.4 |
|  | *Caenorhabditis elegans* | GCF_000002985.6 |
|  | *Lottia gigantea* | GCF_000327385.1 |
|  | *Dictyostelium discoideum* | GCF_000004695.1 |
|  | *Apis mellifera* | GCF_003254395.2 |
|  | *Ciona intestinalis* | GCF_000224145.3 |
|  | *Daphnia pulex* | GCF_021134715.1 |
|  | *Takifugu rubripes* | GCF_901000725.2 |
|  | *Gallus gallus* | GCF_016699485.2 |
|  | *Naegleria gruberi* | GCF_000004985.1 |
|  | *Phytophthora sojae* | GCF_000149755.1 |
| Fungi | *Nadsonia fulvescens* | GCA_001661315.1 |
|  | *Schizosaccharomyces pombe* | GCF_000002945.1 |
|  | *Aspergillus niger* | GCF_000002855.3 |
